# Treatment and Procedures for Ischemic Strokes of Vascular Etiology: Experience of the Eastern Slovakia Stroke Center

**DOI:** 10.7759/cureus.101956

**Published:** 2026-01-20

**Authors:** Martina Zavacká, Jana Pobehová

**Affiliations:** 1 Clinic of Vascular Surgery, East Slovak Institute of Heart and Vascular Diseases (VÚSCH), Košice, SVK

**Keywords:** carotid artery surgery, carotid endarterectomy (cea), ischemic stroke, stroke center, trombectomy

## Abstract

Introduction: A stroke is a condition in which brain cells are damaged or die after blood flow to the brain is interrupted or reduced, which can cause permanent disability or death. More than three-quarters of all strokes are caused by ischemia, and almost a quarter by hemorrhage. It is the second most common cause of death after ischemic heart disease (IHD). The number of affected people is expected to increase to 4.6 million in 2035 due to the ageing of the European population.

Methods: Between 2019 and 2024, 377 patients were treated at the Stroke Center (East Slovak Institute of Heart and Vascular Diseases (VÚSCH)). Of the patients, 62.84% had ischemic stroke due to atherosclerotic arterial disease, 20.42% due to cardiogenic embolization, 15.38% due to combined genesis, and 1.3% due to unusual causes (dissection). The mean age of the patients was 68 (±5) years. The baseline information was improvement/worsening/stability of neurological status according to the National Institutes of Health Stroke Scale (NIHSS) assessment.

Results: The National Institutes of Health Stroke Scale (NIHSS) score on admission, as examined by a neurologist, was 11 (3-20), and on discharge, it had an average of 7 (2-19). Of the patients, 89% (336) showed improvement of neurological status in terms of NIHSS score, 2% (10) deteriorated, and 7.7% (31) had no change.

Conclusion: Early detection and rapid transport, diagnosis, and treatment of patients with stroke in multidisciplinary cooperation (neurologist, radiologist, interventional specialist, and vascular surgeon) are key to improving results. The positive results of stroke centers point to a well-established path in the care of patients with stroke.

## Introduction

A stroke is a condition in which brain cells are damaged or die after the blood flow to the brain is interrupted or reduced, which can cause permanent disability or death. Annually, 1.4 million strokes occur in the European population [1], and 1.1 million people die from it. It is the second most common cause of death after coronary heart disease (CHD) [1]. The number of people affected is expected to increase to 4.6 million in 2035 due to the ageing of the European population [2]. In Slovakia, the incidence is 300/100,000 inhabitants; it is the third most common cause of death. In 2015, 45 billion were spent annually on stroke care in Europe. In the USA, total costs are expected to increase to 129 billion in 2035 (compared to 49.5 billion in 2015) [3]. Of all strokes, 85% are caused by ischemia, and 15% by hemorrhage. The Trial of Org 10172 in Acute Stroke Treatment (TOAST) classification for transitory ischemic attack (TIA)/ischemic stroke includes five categories [4]: (1) large artery atherosclerosis, defined as 50% stenosis or occlusion of an extracranial or intracranial artery; (2) cardioembolic; (3) small vessel occlusion; (4) other etiologies (arteritis and dissection); and (5) undetermined etiology.

In a study by Flaherty et al. (2013) on 2,204 patients with ischemic stroke, large artery atherosclerosis was responsible for 16.6% of strokes. Ipsilateral 50%-99% carotid stenosis was identified in 8%, whereas carotid occlusion or intracranial disease accounted for 3.5% [5]. The proportion of vascular events due to atherosclerotic involvement of large arteries (carotid artery) may be decreasing in connection with the proportional increase in stroke due to cardioembolism [6], which is attributed to a decrease in total cholesterol, low-density lipoprotein (LDL), and blood pressure (BP), an increase in high-density lipoprotein (HDL) cholesterol [7], and a significant increase in the prescription of antiplatelet agents, antihypertensives, and statins [6]. Between 2002 and 2014, there was a 30% decrease in the prevalence of hemodynamically significant (60%-99%) carotid stenosis and a 36% decrease in subtotal (80%-99%) stenosis in patients with TIA/stroke [8], and a significant increase in the prescription of antiplatelet agents, antihypertensives, and statins [6].

## Materials and methods

The East Slovak Institute of Heart and Vascular Diseases (VÚSCH) received the status of a Stroke Center in 2018. It provides continuous procedures: vascular surgery, endovascular mechanical thrombectomy of intracranial arteries (MTE), and acute internal carotid artery (ACI) occlusions from the catchment areas. If stroke or TIA is suspected, the patient should be examined by a neurologist, and after intravenous thrombolysis and diagnostics (brain computed tomography (CT) and CT angiography (CTAG)), the patient should be primarily directed to the nearest Stroke Center. In the six beds of the Neuro Intensive Care Unit, patients are continuously monitored by intensivists and neurologists. Interventions and operations are performed by six certified vascular surgeons and four interventional angiologists. In 2019-2024, 377 patients were treated in our Stroke Center. Of the patients, 62.84% had ischemic stroke due to atherosclerotic arterial disease, 20.42% due to cardiogenic embolization, 15.38% due to combined genesis, and 1.3% due to unusual causes (in all cases, it was dissection). The exclusion criterion was the terminal oncological disorder.

The average age of the patients was 68 (±5) years. Up to 77.6% of the patients suffered from arterial hypertension (often untreated or poorly treated), 48.6% from diabetes mellitus type I and II, 34.5% of the patients underwent cardiac revascularization (coronary artery bypass graft (CABG) and coronary artery stenting) before ischemic stroke, and 35.3% of the patients had a heart rhythm disorder (most often atrial fibrillation, newly diagnosed or inadequately treated, unmasked by ischemic stroke). Peripheral arterial disease (PAD) of the lower extremities was present in 28.6% of the patients, and systemic diseases were noted in 2.9% of the patients. Oncological patients were not suitable for acute closures. The baseline information for us was the improvement/deterioration/stability of the neurological status according to the National Institutes of Health Stroke Scale (NIHSS) assessment (Table 1).

**Table 1:** NIHSS score NIHSS: National Institutes of Health Stroke Scale

Score	Stroke severity
0	No stroke symptoms
1 4	Minor stroke
5-15	Moderate stroke
16-20	Moderate to severe stroke
21-42	Severe stroke

Patients were anticoagulated during the procedure. After carotid artery stenting (CAS), they received dual antiplatelet therapy. After surgical treatment, they received light-molecular-weight heparin (LMWH) (for 10-14 days after surgery) and anticoagulants at long-term intervals.

Statistical analysis

Data were analyzed using descriptive statistical methods. Continuous variables were summarized as mean ± standard deviation (SD) and range, whereas categorical variables were presented as absolute frequencies and percentages. Changes in neurological status between admission and discharge were assessed descriptively using NIHSS score differences. No inferential statistical tests were applied, as the study was designed as a single-center observational analysis focused on descriptive characterization of the treated cohort. Statistical summaries were generated using Microsoft Excel (Microsoft Corp., Redmond, WA).

## Results

NIHSS score on admission, examined by a neurologist, was 11 (3-20), and on discharge, it had an average of 7 (2-19). Of the patients, 89% (336) had improvement of neurological status in terms of NIHSS, 2% (10) deteriorated, and 7.7% (31) had no change. These results confirm the correctness of the establishment of Stroke Centers and emergency care for patients with ischemic stroke or transitory ischemic attack (TIA).

Mechanical thrombectomy of intracranial arteries was performed interventionally in 74.8% (282) of patients. The etiopathogenesis of this subgroup was mainly due to atherosclerotic involvement in 54.25%, cardioembolization in 25.17%, and a combination of both factors in 20%.

Mechanical thrombectomy was performed in the area of the middle cerebral artery (MCA) M2 segment (indication IIb) in 30% (85) of patients: 52.9% (45) in men and 47% (40) in women. In the area of the MCA M1 (indication Ia), it was performed in 26% (73) of patients: 61.6% (45) in men and 38.3% (28) in women. In the area of the MCA M3, it was performed in 8% (23) of patients: 69.5% (16) in men and 30.4% (7) in women.

The posterior cerebral artery (ACP) region was affected in 15% (42) of patients: 57.1% (24) of men and 42.8% (24) of women.

**Figure 1:**
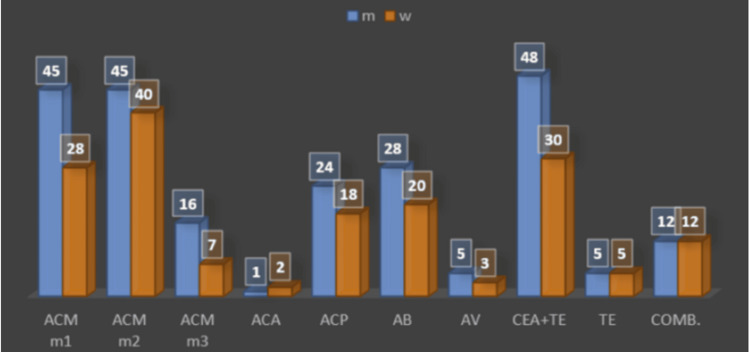
VÚSCH Stroke Center procedures X axis: arterial abbreviations (explained below), Y axis: numbers in the graph express the absolute number of patients ACM m1: cerebral media artery (1 part), ACM m2: cerebral media artery (2 part), ACM m3: cerebral media artery (3 part), ACA: cerebral anterior artery, ACP: cerebral posterior artery, AB: basilar artery, AV: vertebral artery, CEA+TE: carotid endarterectomy+thrombectomy, TE: thrombectomy, comb.: combined procedure, VÚSCH: East Slovak Institute of Heart and Vascular Diseases

The anterior cerebral artery (ACA) region is relatively rarely affected; in our clinical material, it was 1.06% (3) of patients: 33.3% (1) in a man and 66.6% (2) in women. The basilar artery (AB) region was affected in 17% (48) of patients: 58.3% (28) in men and 41.6% (20) in women (Figure 1).

The vertebral artery (AV) was treated in 3% (eight patients): 62.5% (5) in men and 37.5% (3) in women, also considering that AV treatment is not indicated when the AB is patent.

**Figure 2:**
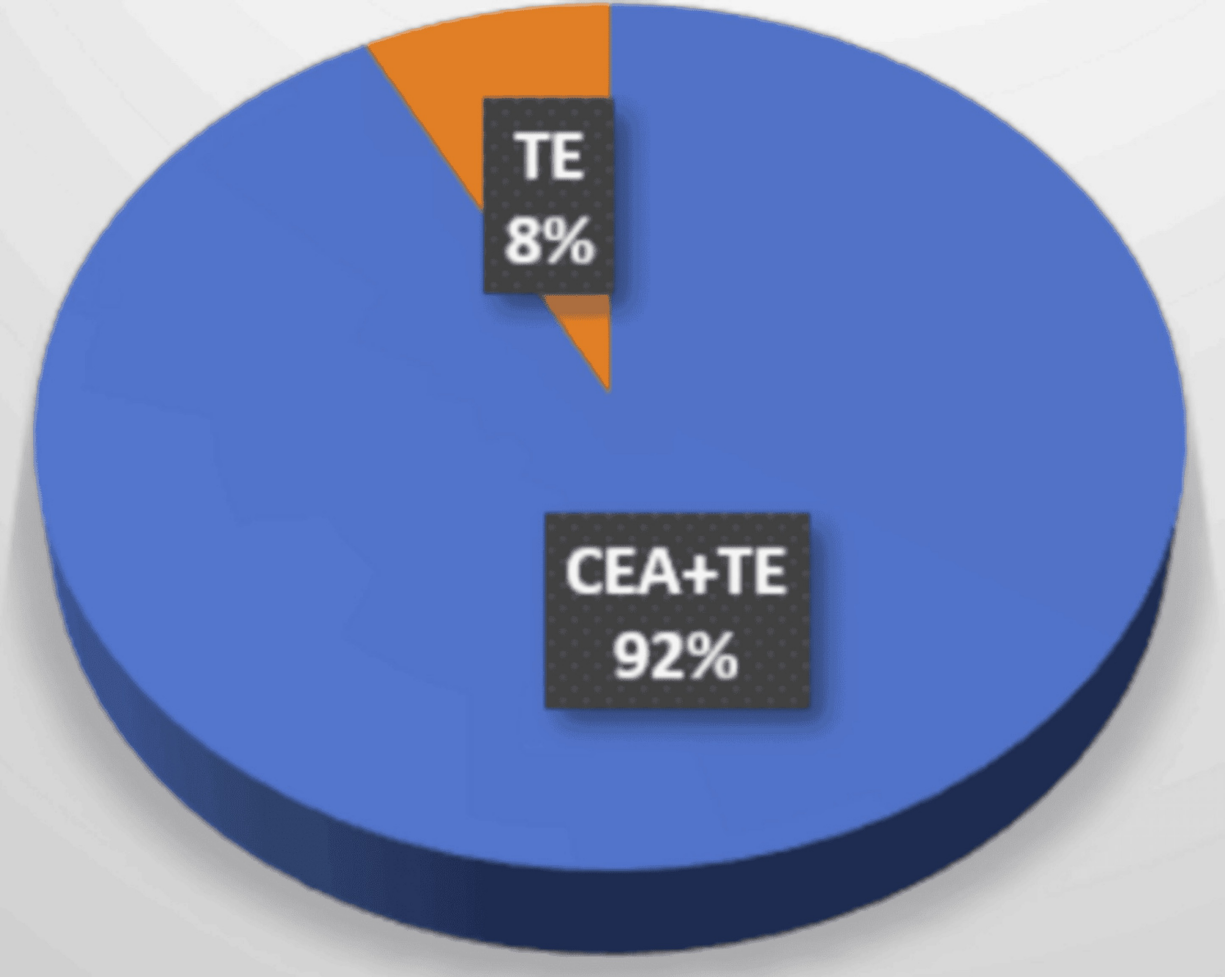
Vascular surgery procedures TE was performed in 8%(6) of patients. CEA+TE was performed in 92% (72) of patients. Orange: TE, blue: CEA+TE TE: thrombectomy, CEA+TE: carotid endarterectomy+thrombectomy

Carotid endarterectomy (CEA) with thrombectomy (TE) of the extracranial section of the internal carotid artery/common carotid artery was performed in 78 patients. Separate thromboembolectomy for acute carotid occlusion was performed in 7.69% (6) of patients: 83.3% (5) in men and 16.6% (1) in a woman. In the vast majority of patients (92.3% (72)), CEA+TE was performed: 66.6% (48) in men and 41.6% (30) in women (Figure 2).

**Figure 3:**
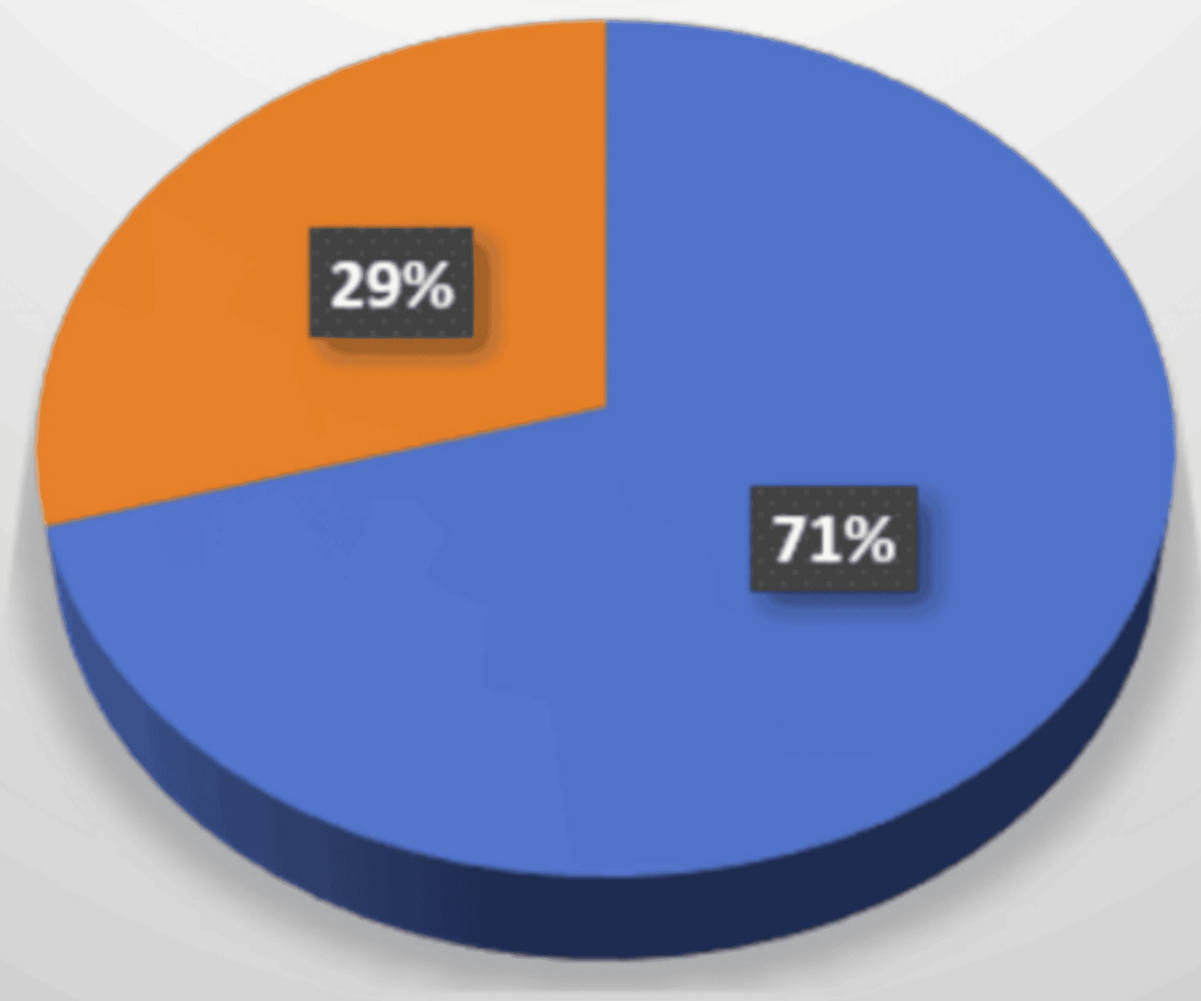
Endovascular + vascular surgery procedures Orange: endovascular + vascular procedures performed in 29% (5) of women. Blue: endovascular + vascular procedures performed in 71% (12) of men.

Combined (hybrid procedure) (CEA+MTE (area ACM M1, ACM M2, ACP)) was performed in 4.7% (17) of patients with acute occlusion of the intracranial and extracranial sections of ICA over five years; 70.58% (12) were in men and 29.4% (5) were in women. Of these, five patients also had artery dissection treated endovascularly (Figure 3).

## Discussion

At our Stroke Center, we follow the latest recommendations of the European Society for Vascular Surgery (ESVS) when treating extracranial and intracranial vessel occlusions.

In acute ICA occlusions in the extracranial segment, stroke in evolution, and crescendo TIA, urgent intervention is required within 24 hours of symptom onset [8-11], which we also follow. In 1.3% of ischemic strokes with symptomatic ICA stenoses, a so-called floating thrombus is present, which is usually attached to the arterial wall (in the AS plaque) [12,13]. It occurs twice as often in men, especially in patients with hypercoagulable status (47%), in patients with thrombophilia, malignancies, infectious diseases, or inflammation, and during pregnancy. It is recommended to start anticoagulant therapy in a therapeutic dose; the administration of thrombolysis is not recommended. On the contrary, an angiosurgical solution is appropriate [13]. In symptomatic ICA stenoses, carotid endarterectomy is recommended within 14 days [14,15]. In our patients, we time it to 7-10 days, depending on the size and extent of the ischemic brain lesion. CEA/CAS is recommended to be performed within six or more days after thrombolysis [16,17]. This period of time is relatively long, and if the patient's neurological status does not improve despite the administration of thrombolysis or if it significantly worsens, we schedule the procedure as soon as possible.

The timing of CEA in international registries has better results within 8 (Sweden) [18], 9 (Germany) [19], and 11 days (Norway, England, and the Netherlands) [20-22]. Meta-analyses from previous years point to the fact that CEA within 48 hours of the onset of neurological symptoms led to a significantly increased 30-day mortality/stroke of up to 11.5%. After 48 hours, the risk was significantly reduced. In high-risk patients with multiple comorbidities, a multidisciplinary team, consisting of a neurologist, interventional specialist, vascular surgeon, and radiologist, decides on the type of procedure [23]. Patients with chronic heart failure (NYHA II-IV), unstable angina pectoris, ischemic heart disease (with even one vascular lesion with stenosis of more than 70%), a myocardial infarction less than 30 days ago, an ejection fraction less than 30%, lung diseases, and kidney diseases are at high risk for CEA.

From an anatomical point of view, patients with ICA involvement just below the base and distally below the clavicle, those who underwent radiation irradiation of the neck, and those with immobility of the cervical spine, contralateral ACI occlusion (which increases the risk of stroke), tracheostomy, and vocal cord paralysis are not suitable for CEA treatment [24-26]. According to the ESVS recommendations, two independent imaging methods before CEA (most often ultrasonography (USG) and CTAG, or USG and MR angiography (MRAG), possibly two independent USG examinations) are required [27]. We only supplement digital subtraction angiography (DSA) in case of discrepancy of results, not routinely, due to the risk of increased embolization into the intracranium [27]. Before CAS, USG and CTAG/MRAG are also required; however, during the therapeutic procedure itself, DSA diagnostics of the aortic arch and the distal branches are also performed [27,28].

Patients with 50%-99% stenosis should be considered for carotid endarterectomy (recommendation IIa and I) [29]. Patients with stenosis less than 50% and patients with chronic occlusions are not recommended for revascularization (III) [29].

Carotid endarterectomy (CEA), either the so-called classic or conventional CEA, is performed with a longitudinal imaging above the ACC and extracranial ICA, with the need to introduce a shunt and maintain flow into the intracranium (Figure 3). It is not possible to perform it in the presence of kinking or coiling of the ACI. The Carotid Stenosis Trialists' Collaboration database verified it as a risk factor for an increase in 30-day stroke [30]. Closure after conventional CEA must be performed with a patch (primary suture is not recommended due to the risk of restenosis) [31-33]; it reduces the incidence of ischemic stroke during 30 days (1.5% versus 4.5%) and the 30-day incidence of ACI thrombosis (0.5% versus 3.1%) [32,33].

In our group of operated patients, we performed the eversion type of endarterectomy, which is our usual practice and achieves excellent treatment results.

Eversion carotid endarterectomy is now the most commonly performed procedure, requiring the separation of the ICA and stripping of the atherosclerotic plaque. It statistically significantly reduces 30-day mortality, stroke, and restenosis [34]. Conversely, ICA is sutured end-to-side and does not require angioplasty. However, when comparing conventional and eversion CEA, no statistically significant differences in 30-day mortality/stroke were found [34].

Carotid endarterectomy should be performed by a vascular surgeon [35-37], either under general or locoregional anesthesia, depending on the experience and choice of the specialist and the patient [30,38-41]. At our workplace, we predominantly use general anesthesia because of its greater experience and comfort for the patient and surgeon.

This study has several limitations that should be considered when interpreting the results. First, it is a retrospective, single-center study conducted at the East Slovak Institute of Heart and Vascular Diseases (VÚSCH), which limits the generalizability of the findings to broader populations. The analysis was based primarily on NIHSS scores at admission and discharge, without including long-term outcomes such as functional recovery (e.g., Modified Rankin Scale), mortality, or quality of life. Furthermore, the short follow-up period only captures neurological improvement at discharge, not long-term prognosis. Selection bias may also be present, as only patients treated at a specialized stroke center were included, potentially skewing the outcomes toward more favorable results.

## Conclusions

Stroke centers throughout the Slovak Republic have shown excellent results in the treatment and rescue of patients with ischemic stroke. Coordination and logistics between the point of first contact and specialized stroke centers still need to be improved. Sufficient equipment with imaging techniques (CT, CTAG, and MRI), examination by a neurologist, and the possibility of administering local thrombolysis allow for a significant improvement in early detection and treatment.

The updated ESVS recommendations clearly regulate and specify the diagnosis, treatment, and management of possible complications that may arise as a result of the treatment of the primary condition. At our workplace, we follow both the ESVS recommendations and our own clinical experience gained from treating patients. Early detection, rapid transport, diagnosis, and treatment of patients with stroke in multidisciplinary cooperation (neurologist, radiologist, interventional specialist, and vascular surgeon) are key to improving results, and the positive results of stroke centers point to a well-established path in the care of patients with stroke.
